# Resting-state EEG recorded with gel-based vs. consumer dry electrodes: spectral characteristics and across-device correlations

**DOI:** 10.3389/fnins.2024.1326139

**Published:** 2024-02-02

**Authors:** Daria Kleeva, Ivan Ninenko, Mikhail A. Lebedev

**Affiliations:** ^1^Vladimir Zelman Center for Neurobiology and Brain Rehabilitation, Skolkovo Institute of Science and Technology, Moscow, Russia; ^2^Institute for Cognitive Neuroscience, HSE University, Moscow, Russia; ^3^MSU Institute for Artificial Intelligence, Lomonosov Moscow State University, Moscow, Russia; ^4^Faculty of Mechanics and Mathematics, Lomonosov Moscow State University, Moscow, Russia; ^5^I. M. Sechenov Institute of Evolutionary Physiology and Biochemistry, Saint Petersburg, Russia

**Keywords:** EEG, dry electrodes, validation, gel-based electrodes, resting-state, signal quality

## Abstract

**Introduction:**

Recordings of electroencephalographic (EEG) rhythms and their analyses have been instrumental in basic neuroscience, clinical diagnostics, and the field of brain-computer interfaces (BCIs). While in the past such measurements have been conducted mostly in laboratory settings, recent advancements in dry electrode technology pave way to a broader range of consumer and medical application because of their greater convenience compared to gel-based electrodes.

**Methods:**

Here we conducted resting-state EEG recordings in two groups of healthy participants using three dry-electrode devices, the PSBD Headband, the PSBD Headphones and the Muse Headband, and one standard gel electrode-based system, the NVX. We examined signal quality for various spatial and spectral ranges which are essential for cognitive monitoring and consumer applications.

**Results:**

Distinctive characteristics of signal quality were found, with the PSBD Headband showing sensitivity in low-frequency ranges and replicating the modulations of delta, theta and alpha power corresponding to the eyes-open and eyes-closed conditions, and the NVX system performing well in capturing high-frequency oscillations. The PSBD Headphones were more prone to low-frequency artifacts compared to the PSBD Headband, yet recorded modulations in the alpha power and had a strong alignment with the NVX at the higher EEG frequencies. The Muse Headband had several limitations in signal quality.

**Discussion:**

We suggest that while dry-electrode technology appears to be appropriate for the EEG rhythm-based applications, the potential benefits of these technologies in terms of ease of use and accessibility should be carefully weighed against the capacity of each given system.

## 1 Introduction

Electroencephalography (EEG) has been instrumental in neuroscientific research, clinical diagnostics, and brain-computer interface (BCI) applications. Conventionally, EEG recordings have relied on gel-based electrodes which necessitate diligent maintenance and often utilize wired amplifiers. However, recent advancements in dry electrode technology have offered a promising alternative, reducing the complexities posed by conventional gel electrodes. These novel dry electrode systems are designed to be user-friendly, opening new possibilities for improving the overall user experience and real-world EEG applications outside the restrictions of laboratory settings (Niso et al., 2023).

The quality of EEG signal acquisition is of paramount importance in any application. Signal integrity directly impacts the accuracy and reliability of EEG-based studies, including cognitive state monitoring, emotion recognition, and motor control research among others. Therefore, rigorous validation and comparison of signal quality between the emerging dry electrode systems and conventional gel electrodes are pivotal for understanding their potential benefits and drawbacks.

Evaluating the quality of resting-state EEG is of particular interest for consumer EEG applications that center around cognitive monitoring. Resting-state EEG data, after processing and analysis, could unveil EEG patterns related to attention, memory, and emotional states (Trejo et al., 2015; Myrden and Chau, 2017; Sun and Yeh, 2017; Katahira et al., 2018; Bajada and Bonello, 2021; Bitner and Le, 2022). Such recordings could allow users to track their cognitive well-being over time, optimize their daily routines, and make informed decisions regarding lifestyle choices that have the potential to impact cognitive performance. Dry-electrode EEG systems could be expanded to include the development of methodologies applicable in real-world situations like detecting fatigue in drivers (Wang et al., 2018, 2020a,b,c; Xu et al., 2021; Chen et al., 2023).

In this study, we conducted resting-state EEG recordings using three modern dry-electrode devices: the PSBD Headband (PSBD LLC, UAE), the PSBD Headphones (PSBD LLC, UAE), and the Muse S Headband (InterAxon LLC, Canada). The approach to device evaluation introduced here could be applied to the other consumer devices in the future. Regarding the devices that we tested, The PSBD systems are novel devices proposed for working with EEG-based paradigms based on EEG spectral characteristics. There have been no previous studies validating the signal quality of the PSBD devices. The Muse Headband, is a widely used commercial device intended for aiding meditation and sleep. Several studies have investigated its signal quality. These studies have shown that, on the one hand, this device captured the typical EEG spectral characteristics, such as the alpha spectral peak. However, on the other hand, it exhibited low reliability in terms of repeatability of measures conducted on different days (Ratti et al., 2017). Additionally, the Muse Headband was susceptible to the influence of artifacts (Przegalinska et al., 2018). Despite certain limitations compared to the medical-grade systems, the Muse Headband has been reported to be effective for ERP research (Krigolson et al., 2017). Additionally this device has found applications in various areas, including predicting task performance (Papakostas et al., 2017), human stress classification (Asif et al., 2019), emotion recognition (Bano et al., 2022), perception of mental stress (Arsalan et al., 2019), and other purposes. Based on these previous reports, we expected the PSBD devices to have somewhat similar advantages and problems in terms of signal quality. Yet, the Muse headband and PSBD systems employ distinct types of dry electrodes. Muse headband is based on gold-coated flat electrodes, while PSBD systems use multi-pin electrodes (called microspikes), which offers advantageous in terms of adaptability to varying scalp contours, more reliable and stable electrical contact, and lower impedance especially in hair-covered areas (Di Flumeri et al., 2019). However, in some implementations these types of electrodes could cause discomfort due to the pressure required for the pin to penetrate the hair and reach the scalp, and possible increase in skin irritation, especially during long-term use.

To serve as a control device representative traditional EEG recordings, we selected the NVX EEG System (MCS, Russia) with gel electrodes, as it fulfilled the necessary criteria for a standard medical EEG recording device.

Consistent with the previous validation studies conducted with the use of other devices (Wyckoff et al., 2015; Ratti et al., 2017; Przegalinska et al., 2018; Radüntz, 2018; Kam et al., 2019; Marini et al., 2019; Cannard et al., 2021), the present study established three measures of signal quality. First, the increase in alpha power was examined for the comparison between the eyes-closed and eyes-open conditions. These two conditions offer an effective basis for comparison, as the alpha band (8–12 Hz) is among the most prominent and readily identifiable frequency bands in the EEG spectrum. Commonly linked with relaxed, wakeful states when the eyes are closed, its prominence and stability facilitate easier identification and analysis. These properties make the alpha band a clear benchmark for evaluating the signal quality of an EEG device. Second, the power correspondence in the standard frequency bands was assessed based on the comparison to the gel-based recordings. Finally, correlation of spectral power between the devices was calculated as a measure of comparison.

## 2 Methods

### 2.1 Participants

Two experiments were conducted. The first experiment compared the performance of the PSBD Headband, the Muse Headband and the NVX in the same subjects. The second experiment compared the PSBD Headphones, the Muse Headband and the NVX. Each experiment involved the separate groups of 15 healthy participants. Concurrent usage of PSBD Headband and PSBD Headphones devices in a single study was not feasible due to the requirements of the NVX setup for varying sensor and reference locations. Yet, some of the recordings exhibited substantial artifacts caused by power-line interference and had to be removed from the analyses. As a result, the first experiment ultimately comprised 11 participants (five males and six females, with an average age of 26.3 ± 5.6 years) and the second experiment included 13 participants (three males and 10 females with an average age of 22.8 ± 1.2years). The participants had normal or corrected-to-normal vision and provided written informed consent, following the guidelines of the ethics protocol approved by the Ethics Committee of Skolkovo Institute of Science and Technology (Protocol No. 9 of Institutional Review Board, June 22, 2022).

### 2.2 Data acquisition

During the experiments, participants were seated comfortably and asked to undergo two phases of EEG recording. The first phase involved a 5-min session with eyes open, followed by an additional 5-min session with eyes closed.

The resting-state recordings were obtained using three different devices in the following sequence: the PSBD device (Headband or Headphones in experiments 1 and 2, respectively), the Muse Headband, and the NVX-36. The PSBD Headband is a soft band placed around the head, equipped with four dry EEG electrodes (T3, T4, O1, O2), each containing 25 pogo pins (spring-loaded electric connectors). The reference and ground electrodes are positioned frontally. The PSBD Headphones come with four dry EEG electrodes positioned at C3, C4, TP9, and TP10. The Muse system uses electrodes located similarly to Fpz, AF7, AF8, TP9, and TP10, with Fpz serving as the reference electrode. In the NVX system, the electrode locations were used matching those of the PSBD Headband (T3, T4, O1, O2) in Experiment 1, and PSBD Headphones (C3, C4, TP9, TP10) in Experiment 2. Fp1-Fp2 electrodes were used for referencing, while ground electrode was placed at Fpz in Experiment 1. In Experiment 2, the reference was set at Cz and the ground electrode was placed at FTT9h. Thus, the choice of reference and ground electrodes in the NVX replicated the settings of the PSBD devices.

During the recordings, the default sampling rates for the portable devices were as follows: 250 Hz for the PSBD devices, 256 Hz for the Muse Headband, and 250 Hz for the NVX system. To ensure optimal signal quality in the recordings, electrode impedance was checked before starting the EEG recording, ensuring that it was below 50 kΩ for NVX and below 500 kΩ for PSBD Headband. As for the Muse Headband, direct access to impedance values was impossible, but the available indirect indicators showed that the impedance level was satisfactory.

### 2.3 EEG processing

The data analyses were performed using MNE Python software (Gramfort et al., 2013) along with other Python libraries. After importing the recorded data into MNE Python, the Muse recordings were resampled at 250 Hz. Subsequently, the data underwent high-pass filtering (>0.5 Hz) using an overlap-add Finite Impulse Response (FIR) filter. To conduct spectral analysis on the time series from each channel, we computed the Power Spectral Density (PSD) using Welch's method (Welch, 1967).

Next, we extracted the mean logarithm of PSD (log PSD) values for several frequency bands at each electrode site for further analysis: delta (1.5–3.5 Hz), theta (4–7.5 Hz), alpha (8–12 Hz), low beta (13–16 Hz), beta (13–21 Hz), high beta (21–32 Hz), and gamma (32–40 Hz).

### 2.4 Statistical analysis

The statistical analyses employed in this study utilized a repeated-measures, within-subject Analysis Of Variance (ANOVA) with participants considered as a random variable and *Device, Condition*, and *Channel group* treated as within-subject factors. Since the portable devices allowed to record activity only from four sensors, we did not examine signal quality in terms of spatial resolution and merged the features within the anterior (T3 and T4 for PSBD Headband and NVX in Experiment 1, C3 and C4 for PSBD Headphones and NVX in Experiment 2, AF7 and AF8 for Muse) and posterior sites (O1 and O2 for PSBD Headband and NVX in Experiment 1, TP9 and TP10 for PSBD Headphones and NVX in Experiment 1 and for Muse in both experiments). In the *post-hoc* pairwise comparisons, *t*-tests were utilized, and a Bonferroni correction was conducted to account for multiple comparisons.

## 3 Results

### 3.1 Experiment 1

#### 3.1.1 Resting-state EEG

**Figure 1:**
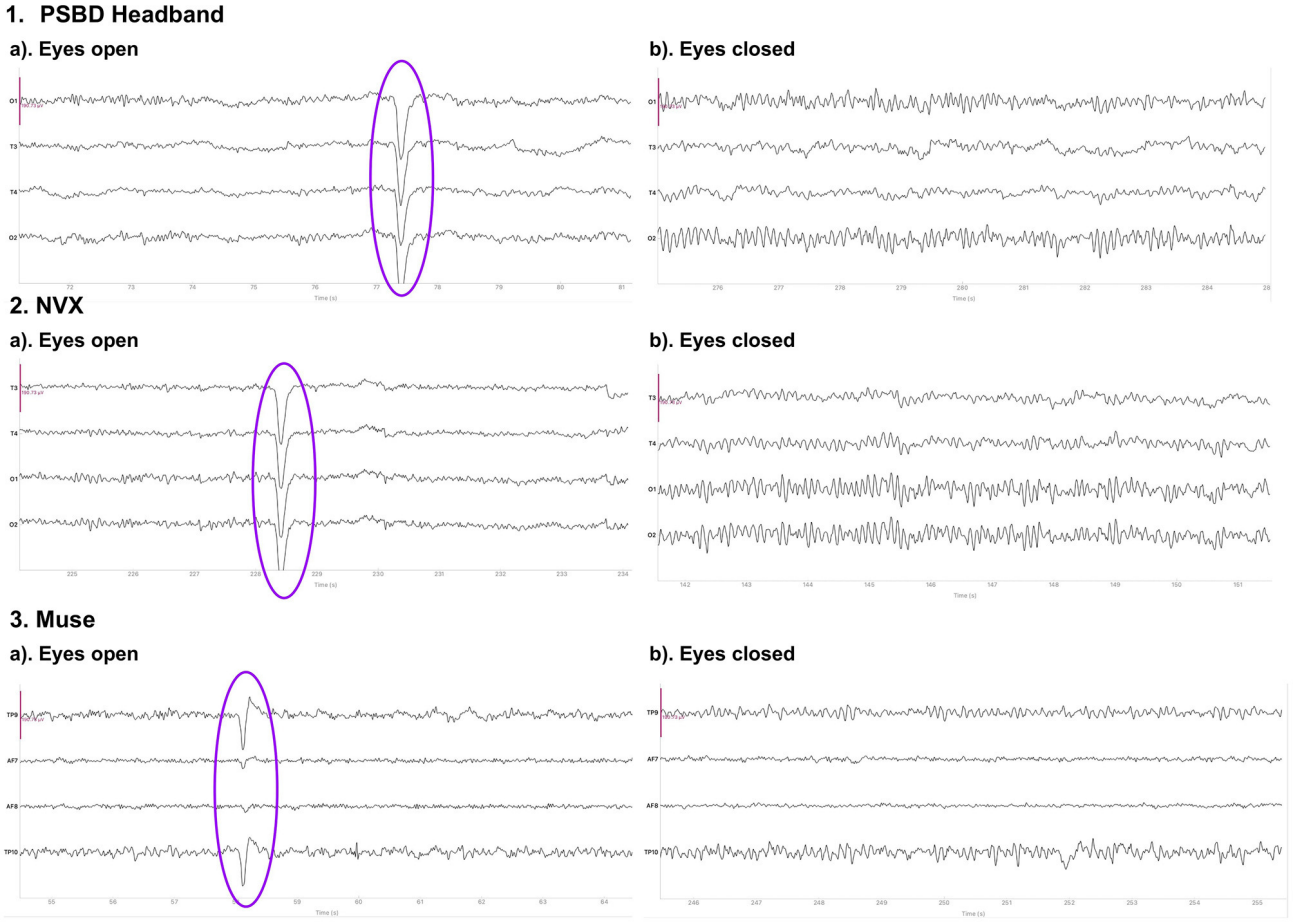
Representative samples of the recordings with the PSBD Headband (1), NVX (2), and Muse (3) devices for the eyes-open and eyes-closed conditions. The purple ovals mark the typical eye-blink artifacts. The *y*-limit is set to ±190.73 mV for all graphs. The signal was bandpass-filtered from 0.5 to 30 Hz for visualization.

Figure 1 depicts the 10-s samples of resting-state EEG data collected in the same subject for the eyes-open and eyes-closed conditions using the three different devices. Upon a visual examination, the presence of alpha spindles is clear during the eyes-closed condition for all three devices. Notably, for the Muse recordings (Figure 1, 3.b), these alpha spindles have a lower amplitude compared to the other devices , which indicates a poorer signal-to-noise ratio that possibly results from the differences in electrode placement compared to the NVX and PSBD Headband.

**Figure 2:**
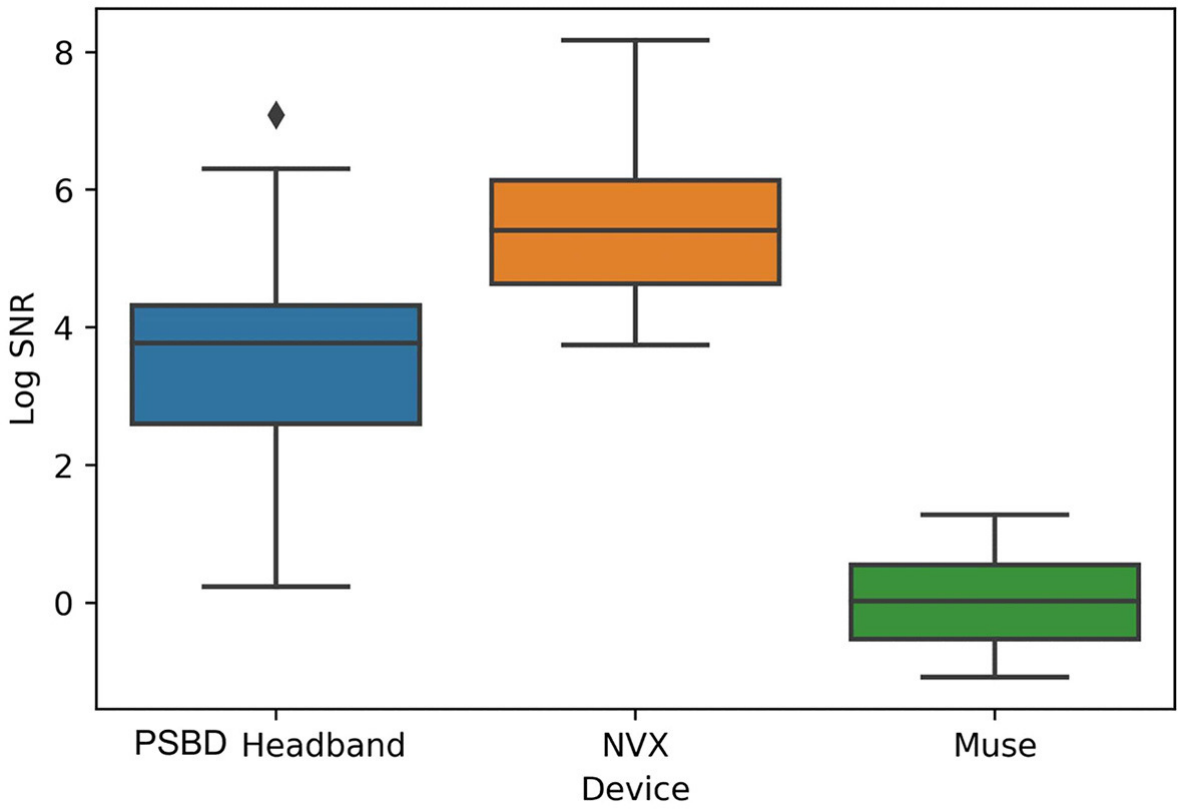
Across-subject (*N* = 11) averages of log SNR demonstrating the differences between PSBD Headband, NVX, and Muse devices.

Physiological artifacts were observed in the recordings from all three devices. However, we chose not to remove them algorithmically, as our aim was to compare the devices in their initial state. To evaluate the contribution of artifacts, we conducted an separate analysis of the signal-to-noise ratio (SNR). We decomposed the signal from the eyes-open condition using Independent Component Analysis (ICA) with the FastICA method, across four channels into four components. We defined SNR as the ratio of the explained variance of the non-artifactual components to the explained variance of the component containing oculomotor artifacts (Figure 2). The analysis showed that the factor of the device was statistically significant: *F*_(20, 2)_ = 37.6201, *p* < 0.0001. In particular, the log SNR of NVX did not differ significantly from the log SNR of the PSBD Headband (*t* = 2.3698, *p* = 0.118) but it was significantly higher than the log SNR of Muse (*t* = 12.631, *p* < 0.001). Additionally, the log SNR of the PSBD Headband was significantly greater than that of Muse (*t* = 5.273, *p* < 0.01).

#### 3.1.2 Spectral analysis

**Figure 3:**
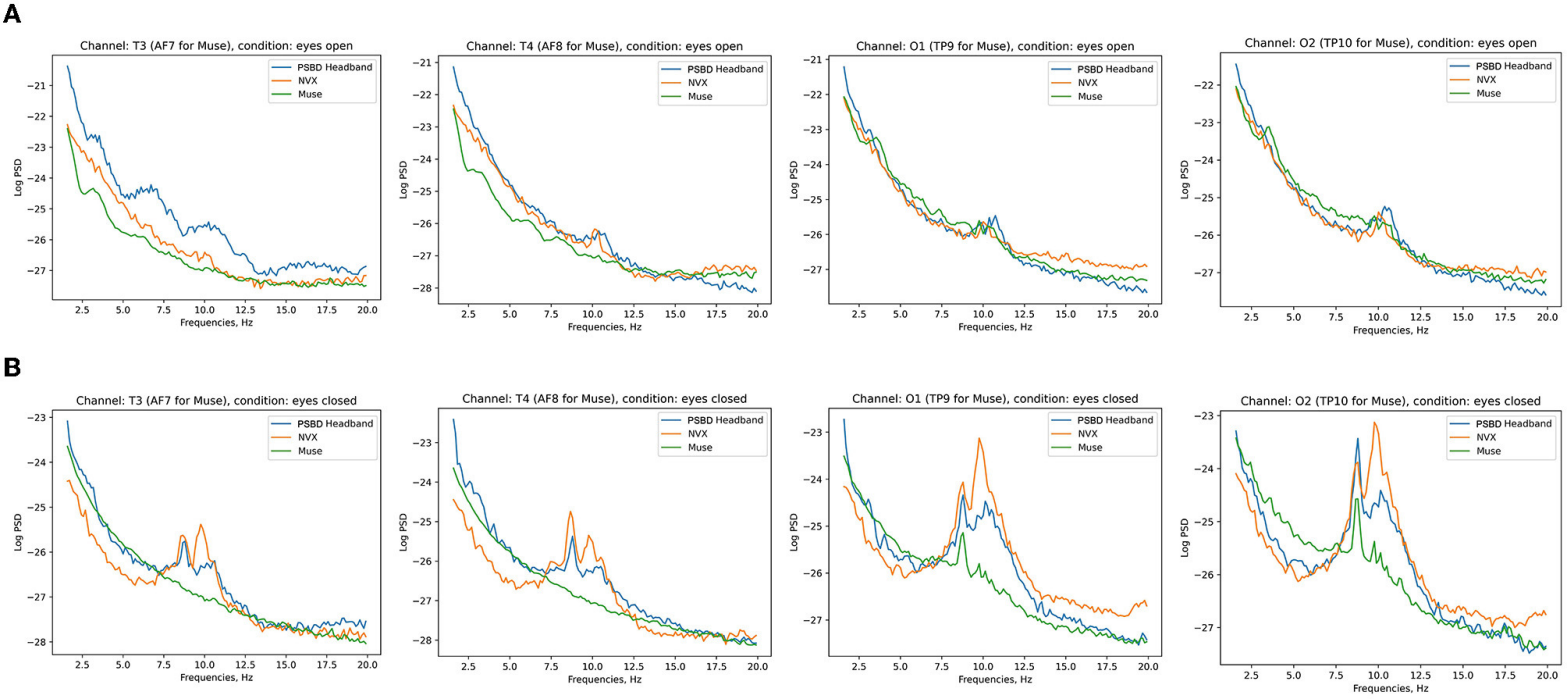
Across-subject (*N* = 11) averages of log PSD demonstrating the difference in alpha power during eyes-open **(A)** vs. eyes-closed **(B)** conditions for PSBD Headband, NVX, and Muse devices.

Figure 3 shows the across-participant average of log PSD, where an increase in alpha power is clear after the eyes were closed.

A repeated measures ANOVA was conducted to examine the effects of device, condition and channel group on the mean value of log PSD for a given frequency range.

**Figure 4:**
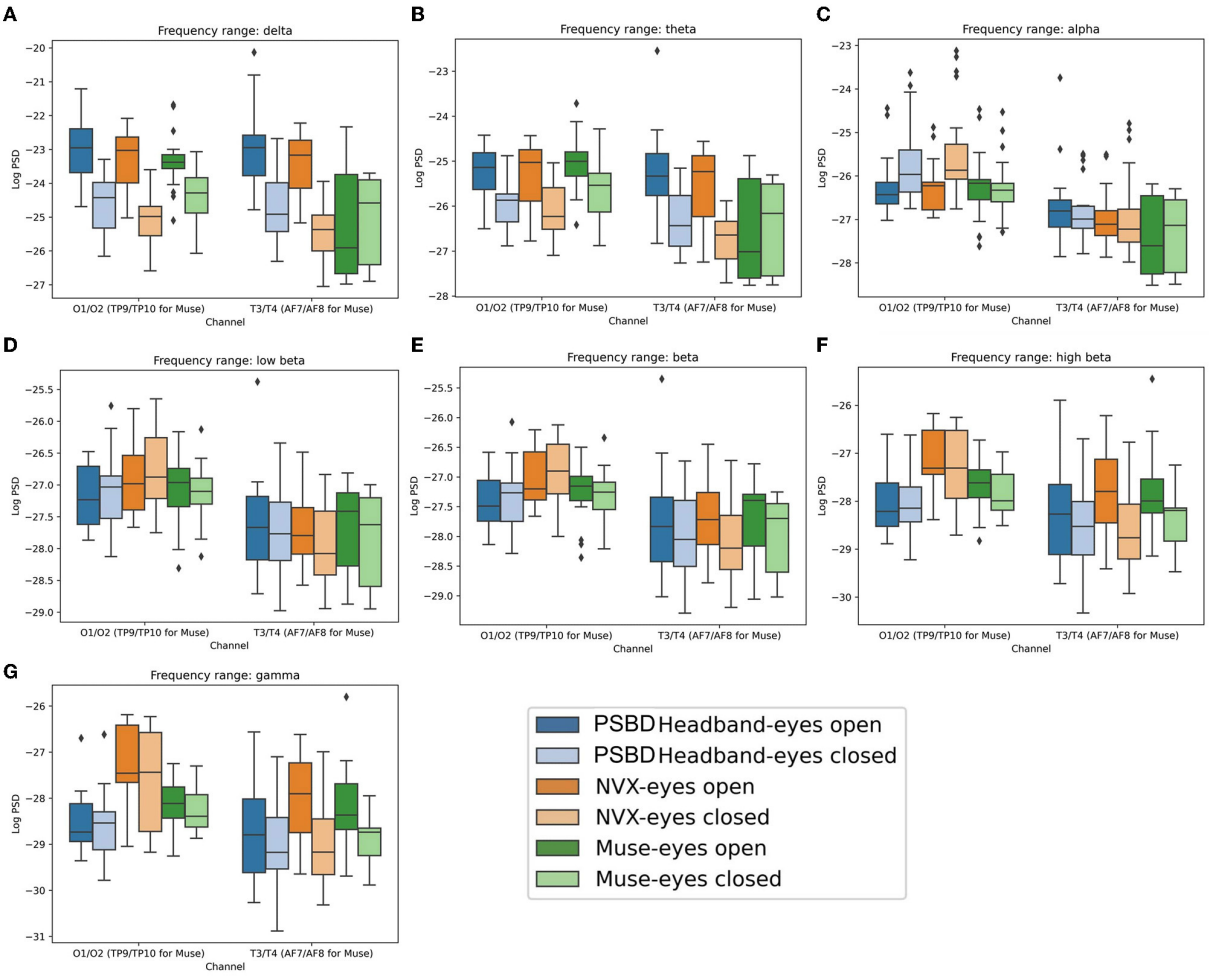
Boxplots showing the distribution of mean log PSD values across conditions and channel groups for different frequency ranges: delta **(A)**, theta **(B)**, alpha **(C)**, low beta **(D)**, beta **(E)**, high beta **(F)**, gamma **(G)** for PSBD Headband, NVX, and Muse.

The results showed that the choice of a device did not have a significant main effect on the delta power [*F*_(2, 20)_ = 2.611, *p* = 0.0983; Figure 4A]. However, both condition [*F*_(1, 10)_ = 63.3793, *p* < 0.001] and channel group [*F*_(1, 10)_ = 27.2044, *p* < 0.001] had significant main effects. Moreover, significant interactions were observed between the device and condition [*F*_(2, 20)_ = 5.3061, *p* < 0.05], device and channel group [*F*_(2, 20)_ = 16.3133, *p* < 0.001], condition and channel group [*F*_(1, 10)_ = 8.9950, *p* < 0.05], as well as device, condition, and channel group [*F*_(2, 20)_ = 9.6415, *p* < 0.05].

The *post-hoc* analysis revealed that when participants had their eyes closed, delta power was significantly reduced compared to the eyes-open condition (*t* = −11.853, *p* < 0.001). Additionally, delta power was stronger for the posterior group of channels than the anterior group (*t* = 5.9207, *p* < 0.001). In the eyes open condition, recordings from the PSBD Headband exhibited higher delta power compared to the NVX recordings (*t* = 2.811, *p* < 0.05) and the Muse recordings (*t*= 4.602, *p* < 0.001). Moreover, the NVX recordings had higher delta power than the Muse recordings (*t*= 3.350, *p* < 0.05). Conversely, during the eyes closed condition, delta power was stronger in the recordings from the PSBD Headband compared to the NVX recordings (*t*= 4.345, *p* < 0.001). However, the NVX recordings had lower delta power than the Muse recordings (*t* = −2.407, *p* < 0.05). Notably, delta power substantially increased after the eyes were opened for both the PSBD Headband recordings (*t* = 12.339, *p* < 0.001) and the NVX recordings (*t* = 17.397, *p* < 0.001).

The delta power was significantly different across the devices and sites. Specifically, the PSBD Headband recordings consistently yielded higher delta power compared to the NVX recordings over both the posterior (*t* = 2.915, *p* < 0.05) and anterior sites (*t* = 4.252, *p* < 0.001). In comparison to Muse, the PSBD Headband recordings had higher delta power only for the anterior sites (*t* = 4.158, *p* < 0.001). Notably, there was a significant decrease in delta power over the anterior sites compared to posterior sites, but this pattern was observed only for the NVX (*t* = −10.932, *p* < 0.001) and Muse recordings (*t* = −6.717, *p* < 0.001).

For the eyes-open condition considered separately, delta power over the anterior sites was stronger for the PSBD Headband recordings as compared to the Muse (*t* = 5.049, *p* < 0.001), and NVX recordings (*t* = 3.950, *p* < 0.05). For the eyes-closed condition, delta power was stronger for the PSBD Headband than for the NVX for both the anterior (*t* = 3.582, *p* < 0.05) and posterior (*t* = 2.511, *p* < 0.05) sites. Furthermore, a statistically significant positive difference in delta power between the eyes-open and eyes-closed conditions was found for the PSBD recordings over the posterior (*t* = 8.828, *p* < 0.001) and anterior sites (*t* = 8.454, *p* < 0.001), for the NVX over the posterior sites (*t* = 11.368, *p* < 0.001) and anterior sites (*t* = 13.206, *p* < 0.001), and for the Muse only over the posterior sites (*t* = 4.484, *p* < 0.05).

The ANOVA analysis conducted for the theta band data (Figure 4B) revealed that there was no significant main effect of the recording device [*F*_(2, 20)_ = 0.6968, *p* = 0.5099]. However, both condition [*F*_(1, 10)_ = 16.2164, *p* < 0.05] and channel group [*F*_(1, 10)_ = 99.6514, *p* < 0.001] had significant main effects. Interactions between device and condition [*F*_(2, 20)_ = 2.9222, *p* = 0.0770] and condition and channel [*F*_(1, 10)_ = 0.1589, *p* = 0.6986] were found to be non-significant. On the other hand, the interactions between device and channel [*F*_(2, 20)_ = 12.9638, *p* < 0.001] and device, condition, and channel [*F*_(2, 20)_ = 10.0947, *p* < 0.001] were statistically significant.

When participants had their eyes closed, theta power was significantly suppressed (*t* = −8.5827, *p* < 0.001). Furthermore, theta power was more prominent over the posterior sites compared to the anterior sites (*t* = 9.4043, *p* < 0.001).

The theta power exhibited distinct patterns across the different recording devices. Specifically, for the PSBD Headband recordings, theta power was lower compared to Muse recordings over the posterior sites (*t* = −2.77, *p* < 0.05), while it was larger at the anterior sites (*t* = 2.875, *p* < 0.05). On the other hand, in the NVX recordings, theta power was suppressed over the posterior sites compared to Muse (*t* = −3.656, *p* < 0.05) and over the anterior sites compared to PSBD (*t* = −3.718, *p* < 0.05). Furthermore, a general tendency for the theta power being lower at the anterior sites compared to the posterior ones was observed for both the NVX (*t* = −10.471, *p* < 0.001) and Muse recordings (*t* = −8.9333, *p* < 0.001).

The theta power had significant differences across the recording devices and eye conditions. When the eyes were open, the PSBD Headband's theta power was significantly higher compared to Muse for the anterior regions (*t* = 3.495, *p* < 0.05). Conversely, the NVX's theta power was significantly lower compared to Muse for the posterior regions when the eyes were closed (*t* = −3.17, *p* < 0.05). Additionally, the theta power of the PSBD Headband recordings was significantly higher compared to the NVX recordings for the anterior regions when the eyes were closed (*t* = 3.387, *p* < 0.05).

Furthermore, when the eyes were open, the theta power over the posterior regions increased for the PSBD Headband (*t* = 5.535, *p* < 0.001), NVX (*t* = 5.785, *p* < 0.001) and Muse (*t* = 3.633, *p* < 0.05). For the anterior channels, this increase was observed only for the PSBD Headband (*t* = 6.313, *p* < 0.001) and NVX (*t* = 8.033, *p* < 0.001).

The ANOVA conducted for the alpha frequency range (Figure 4C) yielded significant main effects of the recording device [*F*_(2, 20)_ = 3.9126, *p* < 0.05] and channel group [*F*_(1, 10)_ = 155.2894, *p* < 0.001]. However, the main effect of condition did not reach significance [*F*_(1, 10)_ = 1.8573, *p* = 0.2028]. The interactions between device and condition [*F*_(2, 20)_ = 1.1401, *p* = 0.3397] and device and channel [*F*_(2, 20)_ = 2.2727, *p* = 0.1290] were also non-significant. In contrast, the interactions between condition and channel group were significant [*F*_(1, 10)_ = 18.1444, *p* < 0.05], as well as the interactions between device, condition, and channel group [*F*_(2, 20)_ = 9.3815, *p* < 0.05].

For the posterior regions, the alpha power was significantly stronger when the eyes were closed compared to when they were open (*t* = −4.295, *p* < 0.001). This effect was statistically significant for the PSBD Headband (*t* = −3.199, *p* < 0.05) and NVX (*t* = −4.739, *p* < 0.001) recordings. Additionally, when the eyes were closed, the PSBD Headband recordings had higher alpha power for the posterior channels than the recordings with Muse (*t* = 5.366, *p* < 0.001), and NVX had higher alpha power for the posterior channels compared to Muse (*t* = 4.855, *p* < 0.001).

The ANOVA analysis conducted for the low beta frequency range (Figure 4D) showed a significant main effect of the channel group [*F*_(1, 10)_ = 85.0479, *p* < 0.001]. The main effects of the recording device [*F*_(2, 20)_ = 0.3708, *p* = 0.6949] and experimental condition [*F*_(1, 10)_ = 0.4008, *p* = 0.5409] were not statistically significant. The interaction between device and condition [*F*_(2, 20)_ = 0.0467, *p* = 0.9544] and the interaction between device, condition, and channel group [*F*_(2, 20)_ = 2.3409, *p* = 0.1220] were not significant either. The only significant interactions were found between device and channel [*F*_(2, 20)_ = 4.7482, *p* < 0.05] and between condition and channel [*F*_(1, 10)_ = 15.2382, *p* < 0.05].

The low-beta power was stronger for the posterior than anterior channels (*t* = 16.0988, *p* < 0.001). Additionally, NVX recordings exhibited stronger low-beta power for the posterior channels as compared to both PSBD Headband (*t* = 4.9032, *p* < 0.001) and Muse (*t* = 2.353, *p* < 0.05).

Similar to the findings for the low beta power, the ANOVA analysis conducted for the beta power (Figure 4E) also revealed a significant main effect of the channel group [*F*_(1, 10)_ = 57.9745, *p* < 0.001]. However, the main effects of the recording device [*F*_(2, 20)_ = 1.5483, *p* = 0.2370] and experimental condition [*F*_(1, 10)_ = 1.2502, *p* = 0.2897] did not reach statistical significance. Likewise, the interactions between device and condition [*F*_(2, 20)_ = 0.2764, *p* = 0.7613] and between device, condition, and channel group [*F*_(2, 20)_ = 2.2954, *p* = 0.1266] were not significant. The only noteworthy interactions were observed between device and channel [*F*_(2, 20)_ = 4.6081, *p* < 0.05] and between condition and channel [*F*_(1, 10)_ = 15.6154, *p* < 0.05].

The beta power was notably higher for the posterior channels compared to the anterior channels (*t* = 13.142, *p* < 0.001). For the anterior channels, the beta power was more stronger during the eyes-open condition compared to the eyes-closed condition (*t* = 3.272, *p* < 0.05). Moreover, the NVX recordings had a stronger beta power for the posterior channels compared to both PSBD Headband (*t* = 6.919, *p* < 0.001) and Muse (*t* = 3.243, *p* < 0.05).

The results from the ANOVA analysis for the high-beta power (Figure 4F) yielded several distinct findings. The main effects of the recording device [*F*_(2, 20)_ = 5.5221, *p* < 0.05], experimental condition [*F*_(1, 10)_ = 8.1142, *p* < 0.05], and channel group [*F*_(1, 10)_ = 22.1371, *p* < 0.001] were all found to be significant. However, the interactions between device and condition [*F*_(2, 20)_ = 1.6315, *p*= 0.2206] and between device, condition, and channel group [*F*_(2, 20)_ = 1.1531, *p*= 0.3358] were not significant. Additionally, the interactions between device and channel group [*F*_(2, 20)_ = 4.9652, *p* < 0.05] and between condition and channel group [*F*_(1, 10)_ = 18.0802, *p* < 0.05] were significant.

During the eyes-closed condition, the high-beta power was significantly suppressed compared to the eyes-open condition (*p* = −5.6774, *p* < 0.001). Conversely, for comparison of the eyes-open condition vs. eyes-closed condition, the high-beta power was higher over the anterior sites (*t* = 5.903, *p* < 0.001). Moreover, the high-beta power was higher over the posterior than anterior channels (*p* = 7.7817, *p* < 0.001).

In terms of the recording devices, the NVX recordings had a higher high-beta power compared to Muse recordings (*t* = 2.4525, *p* < 0.05) and PSBD Headband recordings (*t* = 6.1265, *p* < 0.001). Additionally, the Muse recordings had a elevated high-beta power compared to the PSBD Headband recordings (*t* = 3.137, *p* < 0.001).

Considering the posterior channels specifically, the high beta power was suppressed for the PSBD Headband recordings compared to NVX (*t* = −8.956, *p* < 0.001) and Muse (*t* = −3.795, *p* < 0.001) while it was elevated for the NVX compared to Muse (*t* = 4.036, *p* < 0.001).

The ANOVA analysis conducted for the gamma frequency range (Figure 4G) revealed significant main effects for device [*F*_(2, 20)_ = 7.4974, *p* < 0.05], condition [*F*_(1, 10)_ = 10.4112, *p* < 0.05], and channel group [*F*_(1, 10)_ = 16.4423, *p* < 0.05]. However, the interactions between device and condition [*F*_(2, 20)_ = 2.6102, *p*= 0.0984] and between device, condition, and channel group [*F*_(2, 20)_ = 1.5608, *p*= 0.2345] were not statistically significant. In contrast, the interactions between device and channel [*F*_(2, 20)_ = 4.0474, *p* < 0.05] as well as between condition and channel [*F*_(1, 10)_ = 11.7374, *p* < 0.05] were significant.

Similar to the the findings for high beta, the gamma power was lower when the were closed than when they were open (*t* = −6.5174, *p* < 0.001). This effect was the strongest for the posterior channels (*t* = 6.7493, *p* < 0.001). Yet, the effect was significant only for NVX (*t* = 6.426, *p* < 0.001) and Muse (*t* = 3.906, *p* < 0.001).

Comparing the recording devices, the gamma power recorded by the Muse was lower compared to the NVX recordings (*t* = −3.7136, *p* < 0.05) recordings and higher compared to the PSBD Headband recordings (*t* = 3.2283, *p* < 0.05). Additionally, gamma power was lower for the PSBD Headband as compared to the NVX (*t* = −6.9752, *p* < 0.001).

Considering the signal spatial properties, the posterior channels of the PSBD Headband had lower gamma power compared to both the NVX (*t* = −9.310, *p* < 0.001) and Muse (*t* = −3.913, *p* < 0.001). Conversely, the posterior channels of the NVX had higher gamma power compared to Muse (*t* = 4.595, *p* < 0.001).

#### 3.1.3 Correlation analysis

**Figure 5:**
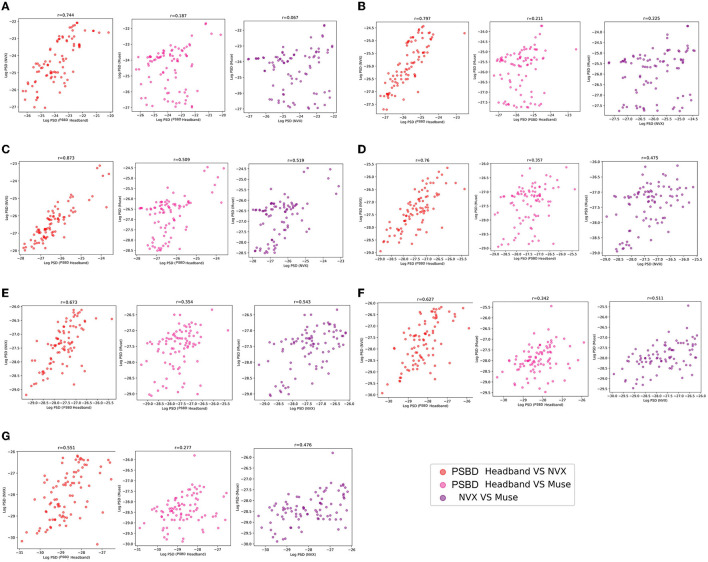
The correspondence between the recordings obtained with different devices (PSBD Headband, NVX, and Muse) assessed as pairwise correlations of mean log PSD values for different frequency range.

The correlation analysis of mean log PSD values revealed that across all frequency ranges, EEG signals of the PSBD Headband matched the conventional EEG recordings obtained with NVX better than those obtained with Muse. Additionally, the correlations between the NVX and Muse were weaker than the correlations observed between the PSBD Headband and NVX (Figure 5).

As it is clear from Table 1, most of the correlation values were found to be statistically significant with the exception for two values for the delta frequency band: the correlation between PSBD Headband and Muse [*r*_(86)_ = 0.187, *p*= 0.081], and the correlation between NVX and Muse [*r*_(86)_ = 0.067, *p*= 0.535]. For all other frequency ranges, the correlations were significant. The correlation was the highest for the comparison of the alpha-range signals of the PSBD Headband and the NVX [*r*_(86)_ = 0.873, *p* < 0.001].

**Table 1:** Results of correlation analysis for PSBD Headband, NVX, and Muse.

**Frequency range/devices**	**PSBD Headband vs. NVX**	**PSBD Headband vs. Muse**	**NVX vs. Muse**
Delta	*r*_(86)_ = 0.744, *p* < 0.001	*r*_(86)_ = 0.187, *p*= 0.081	*r*_(86)_ = 0.067, *p*= 0.535
Theta	*r*_(86)_ = 0.797, *p* < 0.001	*r*_(86)_ = 0.211, *p* < 0.05	*r*_(86)_ = 0.225, *p* < 0.05
Alpha	*r*_(86)_ = 0.873, *p* < 0.001	*r*_(86)_ = 0.509, *p* < 0.001	*r*_(86)_ = 0.519, *p* < 0.001
Low beta	*r*_(86)_ = 0.76, *p* < 0.001	*r*_(86)_ = 0.357, *p* < 0.001	*r*_(86)_ = 0.475, *p* < 0.001
Beta	*r*_(86)_ = 0.673, *p* < 0.001	*r*_(86)_ = 0.354, *p* < 0.001	*r*_(86)_ = 0.543, *p* < 0.001
High beta	*r*_(86)_ = 0.627, *p* < 0.001	*r*_(86)_ = 0.342, *p* < 0.05	*r*_(86)_ = 0.511, *p* < 0.001
Gamma	*r*_(86)_ = 0.551, *p* < 0.001	*r*_(86)_ = 0.277, *p* < 0.05	*r*_(86)_ = 0.476, *p* < 0.001

### 3.2 Experiment 2

#### 3.2.1 Resting-state EEG

**Figure 6:**
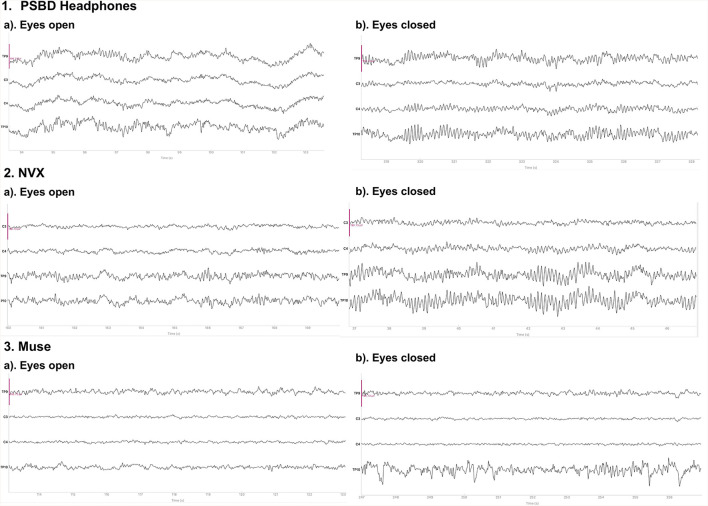
Representative samples of the recordings with the PSBD Headphones (1), NVX (2), and Muse (3) devices for the eyes-open and eyes-closed conditions. The *y*-limit is set to ±190.73 mV in all graphs. The signal was bandpass-filtered from 0.5 to 30 Hz for visualization.

Figure 6 shows the 10-s sample of the resting-state EEG data representative of the eyes-open and eyes-closed conditions. The recordings were conducted in the same participant with the PSBD Headphones, NVX, and Muse Headband. A visual inspection reveals the presence of alpha spindles in both the PSBD Headphones and NVX recordings for the eyes-closed condition. Furthermore, low-frequency drifts are clear in the PSBD Headphones recordings for the eyes-open condition.

#### 3.2.2 Spectral analysis

**Figure 7:**
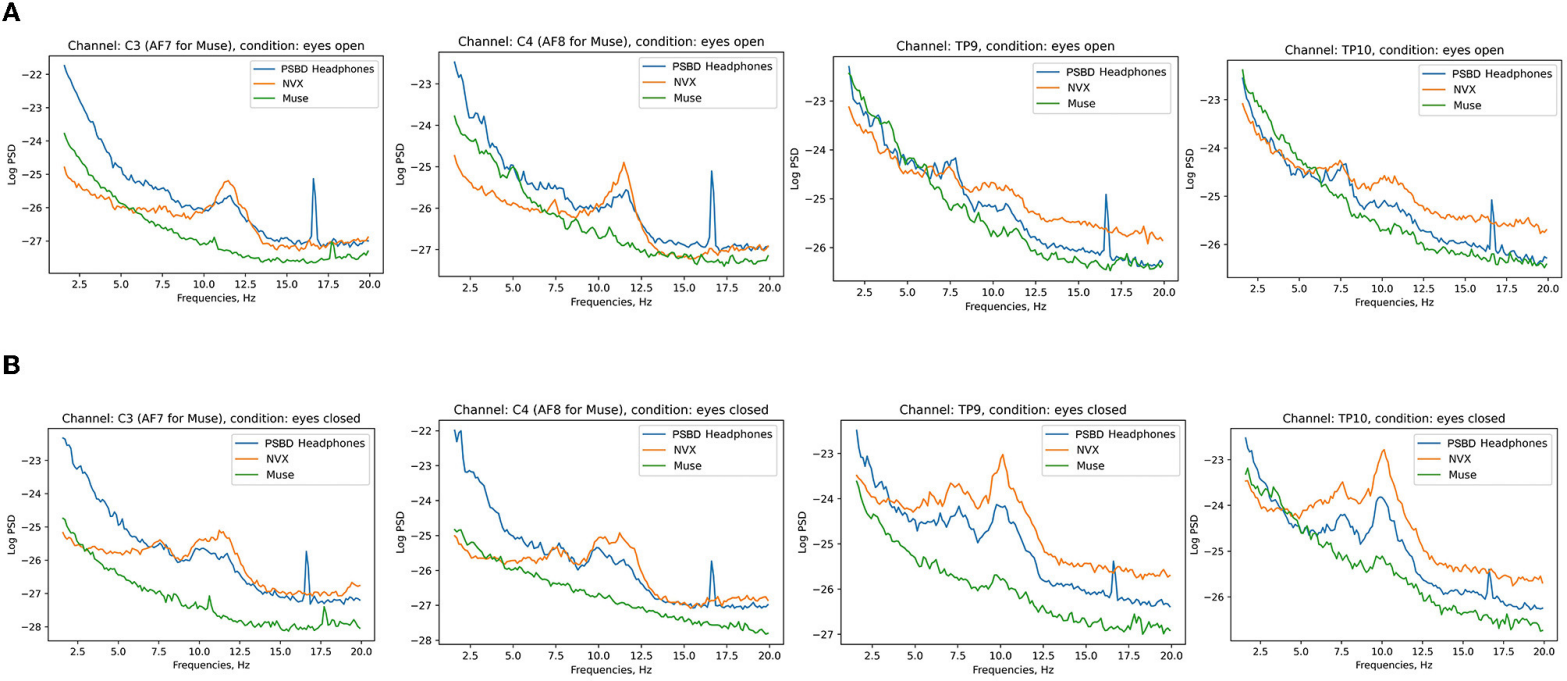
Across-subject (*N* = 13) averages of log PSD demonstrating the difference in alpha power for the eyes-open **(A)** vs. eyes-closed **(B)** conditions for PSBD Headphones, NVX, and Muse.

Figure 7 depicts the across-participant average of log PSD, clearly showing an increase in alpha power when the eyes were closed, especially for the NVX and PSBD Headphones devices.

Like in the Experiment 1, a repeated measures ANOVA investigated the impact of device, condition, and channel group on the mean log PSD within a specified frequency range.

**Figure 8:**
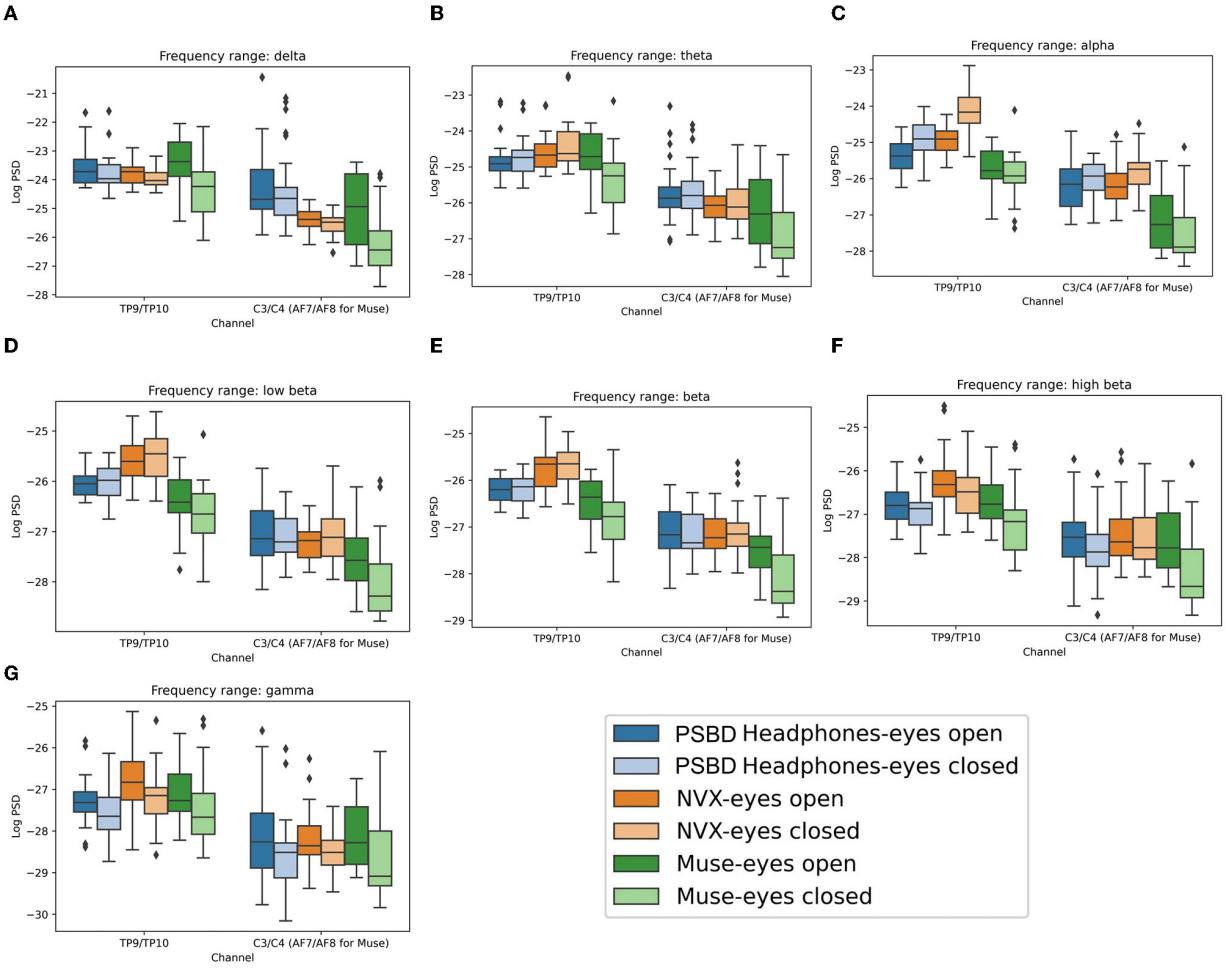
Boxplots showing the distribution of mean log PSD values across conditions and channel groups for different frequency ranges: delta **(A)**, theta **(B)**, alpha **(C)**, low beta **(D)**, beta **(E)**, high beta **(F)**, gamma **(G)** for PSBD Headphones, NVX, and Muse.

There was a significant main effect of device on the delta power [Figure 8A; *F*_(2, 24)_ = 7.684, *p* < 0.05]. Additionally, tmain effects were observed for condition [*F*_(1, 12)_ = 11.665, *p* < 0.05] and channel group [*F*_(1, 12)_ = 115.156, *p* < 0.001]. Additionally, there were significant interactions between device and condition [*F*_(2, 24)_ = 9.437, *p* < 0.001] and between device and channel group [*F*_(2, 24)_ = 18.803, *p* < 0.001]. Conversely, interactions between condition and channel group were not found to be significant [*F*_(1, 12)_ = 0.117, *p*= 0.7385]. The interactions between device, condition, and channel group were also insignificant [*F*_(2, 24)_ = 0.019, *p*= 0.982].

Subsequent *post-hoc* analysis illuminated specific insights. Delta power was significantly lower for both the Muse (*t* = −5.368, *p* < 0.001) and NVX (*t* = −5.9088, *p* < 0.001) devices as compared to the PSBD Headphones. Furthermore, for the eyes-open condition, the delta power had a significant increase (*t* = 5.4733, *p* < 0.001). This increase was observed for the NVX (*t* = 4.965, *p* < 0.001) and Muse (*t* = 5.842, *p* < 0.001).

Notably, for the anterior channels (C3 and C4 for the PSBD Headphones and the NVX, AF7 and AF8 for the Muse), delta power was significantly lower (*t* = −14.7414, *p* < 0.001) than for the posterior channels (TP9 and TP10).

The PSBD Headphones delta power was significantly higher as compared to the NVX, for both the eyes-open (*t* = 3.889, *p* < 0.001) and eyes-closed (*t* = 4.439, *p* < 0.001) conditions. Moreover, the PSBD Headphones delta power was higher than the Muse delta power, but only for the eyes-closed condition (*t* = 5.910, *p* < 0.001).

Specifically for channels C3 and C4, the PSBD Headphones delta power was significantly higher compared to the NVX (*t* = 6.456, *p* < 0.001) and Muse (AF7 and AF8 channels; *t* = 6.180, *p* < 0.001).

In the theta band (Figure 8B), significant effects were observed for device [*F*_(2, 24)_ = 7.135, *p* < 0.05] and channel group [*F*_(1, 12)_ = 263.663, *p* < 0.001]. However, the main effect of condition was not significant [*F*_(1, 12)_ = 2.038, *p* = 0.1789]. Notably, there were significant interactions between device and condition [*F*_(2, 24)_ = 11.363, *p* < 0.001] as well as between device and channel group [*F*_(2, 24)_ = 12.099, *p* < 0.001]. Conversely, the interactions between condition and channel were not significant [*F*_(1, 12)_ = 0.069, *p* = 0.7974], and the interactions involving device, condition, and channel group were also insignificant [*F*_(2, 24)_ = 0.2169, *p* = 0.8066].

*Post-hoc* analysis revealed that the theta power exhibited a significant reduction when comparing the Muse Headband to both the NVX (*t* = −4.951, *p* < 0.001) and the PSBD Headphones (*t* = −5.729, *p* < 0.001). Furthermore, the theta power was significantly lower for the anterior channels (C3 and C4 for the PSBD Headphones and the NVX, and AF7 and AF8 for the Muse) as compared to the posterior channels (TP9 and TP10; *t* = −21.4988, *p* < 0.001).

The decrease in theta power after the eyes were closed was stronger for the Muse Headband as compared to the PSBD Headphones (*t* = −6.845, *p* < 0.001) and the NVX (*t* = −6.559, *p* < 0.001). Additionally, the NVX recordings revealed a significant increase in the theta power after the eyes were closed (*t* = 4.328, *p* < 0.001), whereas the Muse recordings revealed a decrease in theta power after the eyes were closed (*t* = −4.736, *p* < 0.001).

Furthermore, the PSBD Headphones revealed a higher theta power for channels C3 and C4 as compared to the NVX (*t*= 6.456, *p* < 0.001) and Muse (AF7 and AF8 channels; *t* = 6.180, *p* < 0.001).

In the alpha band analysis (Figure 8C), the following effects were significant. Firstly, there was a significant main effect of device [*F*_(2, 24)_ = 89.064, *p* < 0.001] and a significant main effect of channel [*F*_(1, 12)_ = 242.938, *p* < 0.001]. Although the main effect of condition did not reach significance [*F*_(1, 12)_ = 4.475, *p* = 0.056], there was a noticeable trend. Furthermore, interactions were significant between device and condition [*F*_(2, 24)_ = 12.135, *p* < 0.001], device and channel group [*F*_(2, 24)_ = 10.002, *p* < 0.001], and condition and channel group [*F*_(1, 12)_ = 7.0962, *p* < 0.05]. The interaction between device, condition, and channel group was insignificant [*F*_(2, 24)_ = 0.4, *p* = 0.6746].

In the *post-hoc* analysis, the alpha power significantly increased during eyes-closed conditions for the posterior channels TP9 and TP10 (*t* = 4.601, *p* < 0.001). Additionally, this change in the alpha power was significantly lower for the Muse as compared to the NVX (*t* = −15.057, *p* < 0.001) and PSBD (*t* = −12.004, *p* < 0.001). The NVX yielded a significantly higher alpha power as compared to the PSBD Headphones (*t* = 7.2804, *p* < 0.001).

Notably, for the anterior channels (C3 and C4 for the PSBD Headphones and the NVX, AF7 and AF8 for the Muse), the alpha power was significantly lower (*t* = −24.455, *p* < 0.001) than for the posterior channels (TP9 and TP10).

Furthermore, the significant increase in the alpha power after the eyes were closed conditions was observed for the PSBD Headphones (*t* = 4.571, *p* < 0.001) and the NVX (*t* = 8.85, *p* < 0.001) but not for the Muse.

Lastly, the alpha power was stronger anterior over channels (C3 and C4) for the PSBD Headphones (*t* = 10.434, *p* < 0.001) and the NVX (*t* = 9.916, *p* < 0.001) as compared to the Muse (AF7 and AF8 channels). Additionally, the posterior-channel alpha power (TP9 and TP10) alpha power inof the PSBD Headphones was lower compared to the NVX (*t* = −12.185, *p* < 0.001), whereas the Muse had a lower alpha power over these channels as compared to the PSBD Headphones (*t* = −7.134, *p* < 0.001) and the NVX (*t* = 11.508, *p* < 0.001).

In the low-beta frequency range (Figure 8D), significant effects were found for several factors. There was a significant main effect of device [*F*_(2, 24)_ = 27.284, *p* < 0.001]. Additionally, there was a significant main effect of channel group [*F*_(1, 12)_ = 222.06, *p* < 0.001]. However, the main effect of condition was not statistically significant [*F*_(1, 12)_ = 1.64, *p*= 0.2245]. Furthermore, we observed significant interactions between device and condition [*F*_(2, 24)_ = 5.328, *p* < 0.05] as well as between device and channel group [*F*_(2, 24)_ = 6.245, *p* < 0.05]. However, interactions between condition and channel group were insignificant [*F*_(1, 12)_ = 0.3408, *p*= 0.5702]. The interactions between the device, condition, and channel group were insignificant, as well [*F*_(2, 24)_ = 1.275, *p*= 0.2977].

A *post-hoc* analysis revealed specific differences in the low beta power. The Muse exhibited a reduced low-beta power as compared to both the NVX (*t* = −10.267, *p* < 0.001) and the PSBD (*t* = −8.215, *p* < 0.001). The PSBD Headphones also had a lower low beta power as compared to NVX (*t* = −3.9355, *p* < 0.001), particularly for the eyes-closed condition (*t* = −4.121, *p* < 0.001) and the posterior channels (*t* = −9.057, *p* < 0.001). Additionally, the low beta power was consistently lower for the anterior channels (C3 and C4 for the NVX and the PSBD Headphones, and AF7 and AF8 for the Muse) as compared to the posterior channels (TP9 and TP10; *t* = −26.27, *p* < 0.001). The Muse, in particular, had an increased low-beta power when the eyes were open (*t* = 3.788, *p* < 0.05).

In the beta frequency range (Figure 8E), similar statistical patterns emerged. There was a significant main effect of device [*F*_(2, 24)_ = 23.755, *p* < 0.001], a main effect of condition [*F*_(1, 12)_ = 5.663, *p* < 0.05], and a main effect of channel group [*F*_(1, 12)_ = 220.248, *p* < 0.001] on the beta power. Additionally, significant interactions were found between device and condition [*F*_(2, 24)_ = 7.546, *p* < 0.05] and between device and channel group [*F*_(2, 24)_ = 5.611, *p* < 0.05]. However, the interactions between condition and channel group [*F*_(1, 12)_ = 0.753, *p*= 0.4025] and between device, condition, and channel group [*F*_(2, 24)_ = 0.6117, *p*= 0.5507] were not statistically significant.

A *post-hoc* analysis in the beta frequency range revealed that the Muse had reduced beta power compared to both the NVX (*t* = −10.364, *p* < 0.001) and the PSBD Headphones (*t* = −7.551, *p* < 0.001). Additionally, the PSBD Headphones had a lower beta power compared to NVX (*t* = −4.792, *p* < 0.001), particularly over the posterior channels TP9 and TP10 (*t* = −9.663, *p* < 0.001). Moreover, the beta power was significantly higher for the eyes-open condition as compared to the eyes-closed condition (*t* = 3.411, *p* < 0.001). Like the low beta range, the beta power was consistently lower for the anterior channels (C3 and C4 for the NVX and the PSBD Headphones, AF7 and AF8 for Muse) compared to the posterior channels (TP9 and TP10; *t* = −25.275, *p* < 0.001). The Muse had an increased beta power when the eyes were open (*t* = 4.909, *p* < 0.001).

For the high beta frequency range (Figure 8F), we observed significant main effects for device [*F*_(2, 24)_ = 5.5835, *p* < 0.05], condition [*F*_(1, 12)_ = 26.327, *p* < 0.001], and channel group [*F*_(1, 12)_ = 90.719, *p* < 0.001]. Notably, the only significant interaction was between device and condition [*F*_(2, 24)_ = 4.444, *p* < 0.05], while interactions between device and channel group [*F*_(2, 24)_ = 1.666, *p* < 0.2101], between condition and channel group [*F*_(1, 12)_ = 1.412, *p*= 0.2578], and between device, condition, and channel group [*F*_(2, 24)_ = 2.272, *p*= 0.1249] were not statistically significant.

In the *post-hoc* analysis, the NVX had higher high-beta power compared to the Muse (*t* = 6.255, *p* < 0.001) and the PSBD Headphones (*t* = 6.326, *p* < 0.001). During the eyes-open condition, high beta power was stronger than for the eyes closed condition (*t* = 7.148, *p* < 0.001). Additionally, for the eyes-closed condition, the PSBD Headphones exhibited higher high beta power than the Muse (*t* = 3.16, *p* < 0.05). Like the other frequency ranges, the high-beta power was consistently lower for the anterior channels (C3 and C4 for the NVX and the PSBD Headphones, AF7 and AF8 for the Muse) than the posterior channels (TP9 and TP10; *t* = −18.883, *p* < 0.001).

In the gamma-frequency range (Figure 8G), no significant main effect of device was found [*F*_(2, 24)_ = 0.617, *p*= 0.5478]. However, significant main effects were found for both condition [*F*_(1, 12)_ = 45.948, *p* < 0.001] and channel group [*F*_(1, 12)_ = 75.7, *p* < 0.001]. There were no significant interactions between device and condition [*F*_(2, 24)_ = 0.49, *p*= 0.6189], between device and channel group [*F*_(2, 24)_ = 1.7622, *p*= 0.1932], between condition and channel group [*F*_(1, 12)_ = 0.816, *p*= 0.3841], and device, condition, and channel group [*F*_(2, 24)_ = 1.572, *p*= 0.2283].

For the eyes open condition, the gamma power was significantly higher than for the eyes closed condition (*t* = 8.121, *p* < 0.001). Additionally, the gamma power was significantly lower for the anterior channels (C3 and C4 for the NVX and the PSBD Headphones, AF7 and AF8 for the Muse) compared to the posterior channels (TP9 and TP10; *t* = −17.382, *p* < 0.001).

#### 3.2.3 Correlation analysis

**Figure 9:**
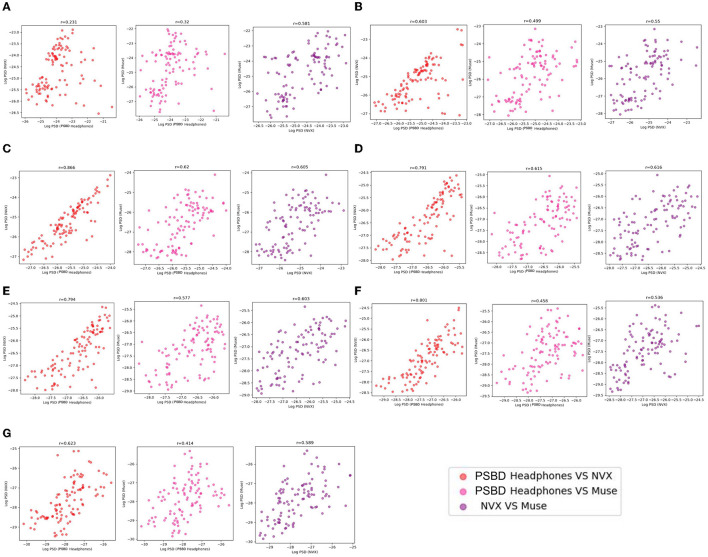
The correspondence between the recordings obtained with different devices (PSBD Headphones, NVX, and Muse) assessed as pairwise correlations of mean log PSD values for different frequency ranges.

The examination of mean log PSD values using the correlation analysis showed that for all frequency ranges except the delta band, EEG signals from the PSBD Headphones closely matched the conventional recordings with the NVX, whereas the correlation with the Muse readings were lower (Figure 9).

As evident from Table 2, the correlation values were statistically significant for all comparisons. Notably, the correspondence between the PSBD Headphones and the NVX was the highests for the alpha frequency range [*r*_(102)_ = 0.866, *p* < 0.001]. For the low-beta, beta, and high beta ranges, the correlation between the PSBD Headphones and the NVX was higher than the correlation between the Muse and the NVX. The correlation values were comparable for the theta and gamma frequency ranges. For the delta frequency range, the correlation between the PSBD Headband and the NVX was considerably lower than the correlation between the NVX and the Muse [*r*_(102)_ = 0.231, *p* < 0.05 vs. *r*_(102)_ = 0.581, *p* < 0.001].

**Table 2:** Results of correlation analysis for PSBD Headphones, NVX, and Muse.

**Frequency range/devices**	**PSBD Headphones vs. NVX**	**PSBD Headband vs. Muse**	**NVX vs. Muse**
Delta	*r*_(102)_ = 0.231, *p* < 0.05	*r*_(102)_ = 0.32, *p* < 0.05	*r*_(102)_ = 0.581, *p* < 0.001
Theta	*r*_(102)_ = 0.603, *p* < 0.001	*r*_(102)_ = 0.499, *p* < 0.001	*r*_(102)_ = 0.55, *p* < 0.001
Alpha	*r*_(102)_ = 0.866, *p* < 0.001	*r*_(102)_ = 0.62, *p* < 0.001	*r*_(102)_ = 0.605, *p* < 0.001
Low beta	*r*_(102)_ = 0.791, *p* < 0.001	*r*_(102)_ = 0.615, *p* < 0.001	*r*_(102)_ = 0.616, *p* < 0.001
Beta	*r*_(102)_ = 0.794, *p* < 0.001	*r*_(102)_ = 0.577, *p* < 0.001	*r*_(102)_ = 0.603, *p* < 0.001
High beta	*r*_(102)_ = 0.801, *p* < 0.001	*r*_(102)_ = 0.458, *p* < 0.001	*r*_(102)_ = 0.536, *p* < 0.001
Gamma	*r*_(102)_ = 0.623, *p* < 0.001	*r*_(102)_ = 0.414, *p* < 0.001	*r*_(102)_ = 0.589, *p* < 0.001

## 4 Discussion

In the present study, we collected resting-state EEG data using several recording devices. Although fairly simple, monitoring resting-state cortical activity has multiple medical and consumer applications, so making these kind of recordings reliable, affordable, and easy to implement offers multiple benefits for the consumers. Therefore, the consumers would benefit from a realistic assessment of the performance of these systems. Here we assessed the recordings obtained with three consumer-grade EEG devices: the PSBD Headband, the PSBD Headphones and the Muse Headband and one medical-grade device, the NVX. We found distinct sets of signal-quality parameters that could be applied to the comparison of different recording devices, illuminating their potential advantages and limitations for various EEG-based applications.

EEG device performance in the delta-rhythm band is of great interest because of the applications where awake, drowsy and sleep states could be assessed. The PSBD Headband consistently had a higher delta power than the other devices. This was particularly clear for the eyes open condition, and for the T3/T4 channels of the PSBD. By contrast, the AF7/AF8 of Muse had a lower delta power. While this finding suggests that the PSBD Headband is suitable for monitoring of low-frequency EEG components (Hinrichs et al., 2020), this frequency range is also prone to low-frequency artifacts which are difficult to account for in consumer settings. Yet, we should not dismiss the low-frequency signal as merely noisy particularly because the PSBD Headband was capable of detecting increases in the delta power (as well as the theta power) during the eyes-open conditions, which aligns with the idea of unstructured processing of the environment (Barry et al., 2007). The same effect was seen for the NVX recordings, which supports the validity of the PSBD Headband's findings. Accordingly, our tests lend credibility to the PSBD Headband's data in the low-frequency range. In Experiment 1, the Muse recordings detected this modulation only over the posterior channels, which questions the reliability of delta-rhythm monitoring with this device.

In contrast to the PSBD Headband, the PSBD Headphones exhibited a greater susceptibility to low-frequency artifacts. The delta power in the recordings from this device was higher compared to the Muse and the NVX. Unlike the Muse and the NVX, the PSBD Headphones delta power was not modulated by opening or closing the eyes.

For the theta-frequency range, our results showed that the PSBD Headband exhibited suppressed theta power compared to the Muse for the posterior channels, while it yielded higher theta power than the Muse for the anterior channels. The NVX system also exhibited unique patterns. In particular, its posterior theta power (O1 and O2) was lower compared to the Muse and its anterior theta power (T3 and T4) was higher compared to the PSBD Headband. These spatial difference could become important for the applications based on the comparison of the theta power over different cortical regions. Notably the PSBD Headband and the NVX detected increased theta power when the eyes were closed, which could be considered a standard spectral change (Barry et al., 2007). On the contrary, while the NVX recorded a notable rise in the theta power over C3, C4, TP9, and TP10, the theta power remained unchanged for the PSBD Headphones regardless of whether the eyes were open or closed.

The alpha frequency range is perhaps the most reliable feature in these kind of recordings because alpha oscillations are typically very prominent and could be easily distinguished from the artifacts. In this frequency range, the Muse device was less sensitive compared to the NVX and the PSBD Headband recordings, which, combined with the consideration of the Muse reduced spatial coverage, suggests that this device does not effectively capture alpha activity, particularly over the posterior brain regions. By contrast, the posterior-alpha rhythm was reliably detected by the PSBD Headband and the NVX. In particular, these devices detected posterior alpha suppression when the eyes were open (Barry et al., 2007). When the eyes were closed and posterior alpha activity was highly synchronized, the PSBD Headband and the NVX system yielded a higher signal amplitude than the Muse Headband. The findings were similar for the PSBD Headphones, where an increase in alpha power occurred when participants closed their eyes, matching the results seen with the NVX. However, it is noteworthy that the overall alpha power was lower for the PSBD Headphones than that the NVX.

The analyses of the low-beta, beta, high-beta and gamma activity over the posterior regions showed that the NVX was consistently more sensitive to these signals compared to both the PSBD Headband and the Muse recordings (Experiment 1). In Experiment 2, the PSBD Headphones had a notably attenuated low-beta, beta, and high beta activity compared to the NVX, particularly over the posterior channels. Moreover, all three dry-electrode devices had increases in the beta power when the eyes were open. The analysis of gamma power, showed that the state of the eyes influenced activity on this range for the NVX and the Muse: gamma activity decreased when the eyes were closed. However, this effect was not observed for the PSBD Headphones.

The correlation analysis dhowed that, across all frequency ranges, the PSBD Headband recordings matched those obtained with the NVX better compared to the Muse. Notably, for the low-beta, beta, high beta, and gamma ranges, the correlation between the PSBD Headphones and the NVX was higher than between the PSBD Headband and the NVX. This phenomenon could be attributed to the PSBD Headphones's electrode placements providing a better sensitivity to detecting high-frequency oscillations. For the theta, alpha, low-beta, beta, and high-beta ranges, the Niery Headphones had a stronger correlations with the NVX than the Muse. However, for the delta range, the correlation between the PSBD Headphones and the NVX was notably low, and for the theta range, it was lower than the correlation between the PSBD Headband and the NVX. Nevertheless, for the alpha range, the correlations were similar between the PSBD Headphones and the NVX and between the PSBD Headband and the NVX. Based on these findings, we conclude that the PSBD dry-electrode technology shows generally matched medical-grade measurements.

Overall, our results suggest that such dry-electrode systems as the PSBD Headband with the channels set to T3, T4, O1, and O2 are appropriate for such consumer application as cognitive monitoring where delta, theta and alpha frequency readings are used to assess the cognitive state. The systems like the PSBD Headphones featuring channels C3, C4, TP9, and TP10 tend to be susceptible to low-frequency artifacts. They are not particularly sensitive to the low-frequency modulations within the theta range, but they are capable of capturing activities within the alpha range. Additionally, they adequately detect modulations in the beta power. Yet, when selecting the system to use, it is essential to consider the specific research objectives and frequency bands of interest. Thus, for the tasks that involve high-frequency oscillations (beta and gamma), the gel electrode-based NVX system is still a more suitable choice due to its higher sensitivity in these frequency ranges. One limitation of the present study is that measurements were made only for the specific electrode configurations available for each device. Accordingly, these specific electrode placements and spatial coverage could have contributed to the observed differences in signal quality across the devices.

In conclusion, our findings highlight the distinct characteristics of signal quality across different recording devices and frequency ranges. The results suggest that the choice of device significantly influences the measurements of EEG power for various frequency bands and brain regions. Researchers and practitioners should carefully consider the specific application and frequency ranges of interest when selecting and recommending appropriate recording devices. The modern dry electrode technology showed promising results, but its performance should be further evaluated based on the specific context and requirements, particularly for such cognitive states as relaxation, stress, and mental workload.

Overall, this study contributes to the growing body of research on the dry electrode technology, its potential applications in EEG research and real-world EEG-based applications, and the development of unified requirements for the consumer-grade applications.

## Data availability statement

The raw data supporting the conclusions of this article will be made available by the authors, without undue reservation.

## Ethics statement

The studies involving humans were approved by Ethics Committee of Skolkovo Institute of Science and Technology. The studies were conducted in accordance with the local legislation and institutional requirements. The participants provided their written informed consent to participate in this study.

## Author contributions

DK: Conceptualization, Formal analysis, Investigation, Methodology, Writing—original draft, Writing—review & editing. IN: Conceptualization, Methodology, Resources, Writing—review & editing. ML: Conceptualization, Supervision, Writing—review & editing.
